# Editorial: Nutritional strategies for enhancing longevity and healthy aging

**DOI:** 10.3389/fragi.2026.1898942

**Published:** 2026-08-18

**Authors:** Pintu Choudhary, Pratik S. Gaikwad, Harsh B. Jadhav

**Affiliations:** 1 Department of Food Technology, College of Agricultural Engineering and Technology Dr. Rajendra Prasad Central Agricultural University, Samastipur, India; 2 School of Biotechnology and Bioinformatics, D Y Patil Deemed to be University, Navi Mumbai, India; 3 Food Technology, Amity Institute of Biotechnology, Amity University, Rajasthan, Jaipur, India

**Keywords:** ageing, antioxidant, lifestyle strategies, nutrients, phytochemicals, polyphenols

## Introduction

Aging is one of the most significant social changes of the 21st century, with a decrease in the number of young individuals and an increase in the number of older individuals (Gianfredi et al., 2025; Ismail et al., 2021). The population over 60 years of age is expected to exceed 2.1 billion by the end of 2050, accounting for 21% of the world’s total population (United Nations, 2023). Aging is an unavoidable physiological process that causes a decrease in biological functions; however, aging does not necessarily refer to illness (Noto, 2023). In recent years, nutritional strategies have attracted attention because they increase life expectancy and keep aging individuals free from disease (Hu, 2023). A well-planned diet plays a crucial role in preventing the onset of age-related medical conditions such as dementia, cardiovascular disease, and neurodegenerative disease while maintaining good mental health. Dietary patterns change with age. A diet suitable for an individual in their 40s may not be a good choice for the same individual in their 60s (Fekete et al., 2023). The calorie requirements of an individual vary with aging. For example, the calorie requirements of a man aged 60–69 are 2,000 kcal/day and 2,900 kcal/day for a sedentary and active lifestyle, respectively (Fekete et al., 2023). In addition to daily calorie intake, the macro- and micronutrients present in the diet are also very important (Faria-Silva et al., 2020). This Research Topic aims to delve into various nutritional interventions that can slow the aging process, promote healthier aging, and reduce the incidence of age-related diseases. Its goal is to determine how specific nutrients, dietary patterns, and lifestyle choices influence aspects of aging, such as cellular senescence, oxidative stress, inflammation, and mitochondrial health. The contributions to this Research Topic identified effective nutritional strategies that promote overall health and extend the health and lifespan of elderly populations. This includes the effects of anti-aging foods, plant-based diets, phytochemicals, and antioxidants, in addition to probiotics and prebiotics.

## The importance of nutritional strategies

Unhealthy eating patterns/unhealthy diets are affecting consumers aged over 60. There are many studies published in the literature showing the importance of a proper diet for healthy aging and to prevent lifestyle-related diseases. The quality of a diet depends on the macro- and micronutrients present in the food, and a diet can be quantitatively characterized by the calories it supplies. The intake of major food components, such as lipids, proteins, and carbohydrates, changes with increasing age. In total, 50%–60% of daily energy should come from carbohydrates, followed by 25%–30% from lipids (Fekete et al., 2023). Consumption of saturated/animal fats should be avoided, and vegetable oil should be used as a cooking medium instead. Previous studies have suggested a close association between the consumption of animal fats and the risk of type 2 diabetes (Neuenschwander et al., 2020). Reducing the intake of saturated fats also lowers the risk of cardiovascular disease by reducing low-density lipoproteins. Aging populations are at a higher risk of osteoporosis and sarcopenia, which cause loss of physical strength. Increasing protein intake through sources such as seafood, meat, milk, and dairy products can slow down the loss of lean muscle mass associated with sarcopenia. The recommended daily intake of protein is 1–1.2 g/kg/day for healthy aging and 1.2–1.5 g/kg/day for individuals with chronic conditions (Harris et al., 2025). The daily dietary fiber requirement (30–40 g) can be met by consuming fruits and vegetables. 1n addition to major food components, minor food components (vitamins and minerals) also play a key role in healthy aging. A daily intake of 1,200 mg of calcium is recommended for men over 70 and women over 50 to prevent the risk of osteoporotic fractures. Individuals should meet their daily vitamin intake requirements to prevent age-related diseases and promote healthy aging.

## Final remarks

The present Research Topic has published a total of 9 original research and review articles focusing on the importance of diet and nutritional supplements for healthy aging. Tian and Chen (2026) reported on the use of nutritional supplements by 9,986 participants with chronic diseases aged 65 and above in China. The authors reported that approximately 1,189 individuals used nutritional supplements, with calcium being the most commonly used, followed by multivitamins and protein supplements. A review by Zhang et al. (2025) highlighted the importance of dietary phenolics and exercise supplementation in delaying the aging process. The authors noted that mitochondrial dysfunction and the buildup of ROS are responsible for aging. However, these are caused by individuals’ lifestyles, genes, and age. Phenolic compounds and exercise have a positive effect on mitochondria, which helps delay the aging process and is consistent with the existing concept of anti-aging. The synergistic effect of exercise and phenolics enhances mitochondrial health through biogenesis and antioxidant defenses. This effect balances cellular oxidative stress and improves energy production.

A U.S. study was carried out by Li et al. (2025) based on data obtained from the National Health and Nutrition Examination Survey (NHANES) from 2007 to 2010 to 2015–2018. The authors reported on the effects of the dietary inflammatory index and vigorous-intensity exercise on phenotypic age acceleration in 4,167 adults. The researchers concluded that regular consumption of an anti-inflammatory diet and reduced levels of vigorous-intensity exercise were closely related to a delay in biological aging among U.S. adults. Another study, by Tong et al. (2025), also used data from NHANES (1999–2018) to evaluate the effect of serum sodium levels on the biological aging process in adults from the United States. Their article included 18,301 participants over the age of 20. The authors concluded that the biological aging process in adults can be delayed by maintaining an optimal serum sodium level between 138 and 142 mmol/L. For adults with diabetes, hydration plays a key role in healthy aging.

The promising results of consuming a *Phaeodactylum tricornutum* microalgae supplement by 66 individuals aged 55–75 years with memory impairment were reported by Goodbody et al. (2025). Their study reported this microalgae’s beneficial effects on immune markers and cognitive function. Jia et al. (2025) conducted an analysis of NHANES data from 2005 to 2008 on individuals over 40 years old with age-related macular degeneration (AMD) pathogenesis and systemic inflammatory response index (SIRI). The authors reported that a higher SIRI score alone is responsible for a higher AMD risk in U.S. individuals, and it may be used as a biomarker for identifying high-risk subjects.

In 2019, the EAT-Lancet Commission proposed a unique dietary pattern known as the Planetary Health Diet (PHD). A study by Huang et al. investigated the relationship between the PHD Index and biological aging among individuals from the United States. The study considered 44,925 individuals for whom data were obtained from the National Health and Nutrition Examination Survey (NHANES) from 1999 to 2018. The authors reported the existence of a significant relationship, showing that strictly following the PHD may delay biological aging and impact longevity. The biological aging process can also be delayed by dietary diversity, as reported by Liao and Li (2024). The results of their study provide significant evidence of a correlation between higher dietary diversity and delayed phenotypic age. Similarly, the review by Giraldo-Castrillon et al. (2026) highlighted the effect of consuming milk and milk products on the cognitive function of older adults. Dairy products are considered neuroprotective due to the presence of bioactive components and higher nutrient density, making them a potential nutritional strategy for older individuals to preserve cognitive function.

In conclusion, the studies published in this Research Topic highlight the importance of nutritional strategies in delaying the process of biological aging in individuals across different age groups.

Delaying aging was found to be closely related to dietary diversity, a planetary health diet, microalgae supplementation, and intake of dairy products, each of which has its own mechanism to prevent age-related diseases.
